# Supplementary material for: Recognising and responding to the community needs of gay and bisexual men around mpox
Source: PLoS One. 2024 Nov 12;19(11):e0313325. doi: 10.1371/journal.pone.0313325 (PMC11556694; doi:10.1371/journal.pone.0313325)
Supplement: S1 Appendix — (DOCX) [file pone.0313325.s001.docx]

# Appendix 1. Survey Questions

Branching Question

● Have you had monkeypox?

○ No

○ Yes, I had/currently have monkeypox

Open-ended Questions

1) [If branching question = Yes] Please tell us about your experience of having had monkeypox

● You might want to describe experiences related to:

realising you might have monkeypox, interactions with, and care offered by, healthcare professionals, discussing your diagnosis with others, self-isolation , recovery

1) Please tell us about the impact (if any) the monkeypox public health emergency has had on your daily life.

The impact on your life might be big or it might be small, or it may not have impacted you at all. Please feel free to share any thoughts you have in relation to any domain of your life.

2) Please tell us about any worries or concerns you have around preventing monkeypox?

You might want to describe your thoughts and/our feelings related to preventing yourself from getting monkeypox, preventing monkeypox among your friends or lovers; or any wider issues around the transmission of monkeypox

3) What are your thoughts about how monkeypox has been managed in Ireland so far? Please explain your answer in detail.

You might want to describe your thoughts on how the government, health services, community organisations etc have managed the prevention of monkeypox.

You might also have thoughts on the type of care or support available for those who get or have had monkeypox.

4) What kind of information and/or support would be helpful to you in relation to the current monkeypox public health emergency?

Please share any thoughts on the different types of information and/or support you think would be helpful; and who would/should provide this information/support (e.g government, healthcare organisations, community organisations or from friends/family or the wider public etc.). This can be any kind of support, not just healthcare related support, which would make the prevention or experience of monkeypox easier.

5) Is there anything else you would like to add about monkeypox or the response to monkeypox?

Quantitative Questions

How concerned are you about getting monkeypox?

● 1-5 I am extremely concerned about getting monkeypox - I am not concerned at all about getting monkeypox

How concerned are you about getting other STIs?

● 1-5 I am extremely concerned about getting other STIs - I am not concerned at all about getting other STIs

How ‘at-risk’ of getting monkeypox do you think you are?

● 1-5 It is extremely unlikely that I will get monkeypox to It is extremely likely that I will get monkeypox . Incl don’t know.

How ‘at-risk’ of getting other STIs do you think you are?

● 1-5 It is extremely unlikely that I will get other STIs to It is extremely likely that I will get Other STIs . Incl don’t know.

How much negative impact would getting monkeypox have on your daily life?

● 1-5 Extremely negative impact to no negative impact

OR if participant has already had monkeypox

How much negative impact has having monkeypox had on your life?

● 1-5 Extremely negative impact to no negative impact

How informed are you about the current monkeypox public health emergency?

● 1-5 I have heard/read a lot about monkeypox to I have heard/read very little about monkeypox incl. I do not know about it

How would you rate your understanding of the public health guidance regarding monkeypox?

● 1-5 I understand the current public health information regarding monkeypox very well to I do not understand the current public health information regarding monkeypox

Where have you received information on the current monkeypox public health emergency?

● Irish TV/Radio

● International TV/Radio

● Irish government sources (HSE, Dept of Health)

● Irish community organisation resources (MPOWER, Man2Man)

● From a healthcare professional at a GP clinic

● From a healthcare professional at a hospital

● From a healthcare professional at a pharmacy

● From a healthcare professional at a sexual health clinic

● International government sources

● Twitter Posts (Non governmental/health service)

● Facebook Posts (Non governmental/health service)

● Instagram Posts (Non governmental/health service)

● TikTok Posts (Non governmental/health service)

● Directly from friends

● Community Outreach Workers

● Hookup Apps

● Other

How trustworthy do you find information on the current monkeypox public health emergency which comes from (above list)

● 1-5 I would find it extremely trustworthy to I would find it extremely untrustworthy

Demographic Questions

○ age

○ gender

○ sexual orientation

○ ethnicity

○ country of birth

○ highest educational qualification

○ employment status

○ income

○ city & county of residence
