# Supplementary material for: Recognising and responding to the community needs of gay and bisexual men around mpox
Source: PLoS One. 2024 Nov 12;19(11):e0313325. doi: 10.1371/journal.pone.0313325 (PMC11556694; doi:10.1371/journal.pone.0313325)
Supplement: Attachment — Submitted filename: Review report PONE-D-24-32402.docx [file pone.0313325.s002.docx]

Although your article (PONE-D-24-32402) is organised effectively, there are a few places where it could be more focused, succinct, and clear. Based on the overall composition, the following particular suggestions will improve your document: I recommend this manuscript after minor revision.

1. Consistency in Title and Terminology:

• Ensure that the language is consistent. As an illustration, you utilise both "mpox" and "mbpox." Stick to a single term throughout the entire document. Per the most recent WHO standards, the recommended term is "mpox".

2. **Context**: Explain why the mpox outbreak in 2022 worried gbMSM populations especially. This should include a succinct description of the virus's characteristics, the reasons certain communities were disproportionately afflicted, and any early opinions or responses from the general public.

**Relevance**: Highlight why the study is specifically focused on Ireland. Is it due to a unique public health response, or were there specific community dynamics in play?

**3. Methods**

- **Detail the Methods**: You could provide a little more detail to the description of your mixed-methods approach, like the duration of the survey, the participant recruitment process, and any analysis tools that were employed.
- **Reflexive Thematic Analysis**: Mention briefly what "reflexive thematic analysis informed by critical realism" entails, especially for readers who may not be familiar with these concepts.

**4. Findings**

- **Contextualize the Numbers**: While stating that 163 men participated, it could be useful to provide some context, like how representative this sample is of the wider gbMSM community in Ireland.
- **Thematic Overview**: You’ve listed the four themes developed from the qualitative data. Consider briefly explaining what each theme entails or its significance rather than just listing them. This gives the reader a better grasp of the findings.

**5. Discussion**

- **Strengthen the Argument**: Although you discuss the efficacy of community-led projects, it would be helpful to give specific instances or further describe these activities. This would support your claim about the significance of these kinds of projects.
- **Institutional Critique**: Any particular instances from the research or literature that highlight the negative effects of institutionalised homophobia on public health outcomes could be added to the conversation about the issue.
- **Balance the Discussion**: In addition to stressing the difficulties, it could be a good idea to highlight any achievements or compliments received on the public health response, if any. This makes the conversation more fair-minded.

**6. Conclusion**

- **Actionable Recommendations**: Your conclusion is strong, but it would benefit from more actionable recommendations. For instance, how can public health strategies are made more inclusive and transparent? What specific steps can be taken to ensure equitable access and address structural inequalities?

**7. Language & Tone**

- **Conciseness**: There are some opportunities to tighten the language. For example, "the study underscores the need for inclusive, culturally sensitive, and transparent public health strategies" could be simplified to "the study highlights the need for inclusive and transparent public health strategies."
- **Avoid Jargon**: Some readers may not be familiar with terms like "critical realism" or "reflexive thematic analysis." While these are important, consider simplifying or explaining them briefly to ensure accessibility.

**8. Overall Structure**

- **Flow & Transitions**: Ensure smooth transitions between sections. For example, the findings section should naturally lead into the discussion, where the implications of these findings are explored in more depth.

Incorporating these improvements should make your write-up clearer, more persuasive, and accessible to a broader audience.
