# Supplementary material for: Recognising and responding to the community needs of gay and bisexual men around mpox
Source: PLoS One. 2024 Nov 12;19(11):e0313325. doi: 10.1371/journal.pone.0313325 (PMC11556694; doi:10.1371/journal.pone.0313325)
Supplement: Attachment — Submitted filename: Response to reviewers.doc [file pone.0313325.s003.doc]

20^th^ September 2024

Professor Emily Chenette

Editor-in-Chief

PLOS ONE

Dear Professor Chenette,

I wish to thank you, your editorial team and the reviewers for the helpful recommendations in strengthening our submission. Below I detail how the suggested revisions have been made.

**Editorial Revisions**

1. We have addressed the stylistic errors related to author information and the wider document.
2. We have made the specified changes to our ethics statement and replicated the statement which is on the system into the revised document as suggested.
3. We have ensured that the funding information and financial disclosure are in line.
4. We specified that the funders had no role in study design, data collection and analysis, decision to publish, or preparation of the manuscript.
5. We have clarified that the version of findings published on the HIV Ireland website is not peer reviewed and presents an overview of the work, rather than an academic publication and therefor is not dual publication.
6. We have provided details of the contact information for the ethics committee for data access requests. As the data could be potentially identifiable it is not publicly available.
7. As per point 2.

**Reviewer 1**

1. We have addressed the stylistic inconsistencies and errors throughout the manuscript as advised
2. We have given some further detail on why this outbreak was particularly of concern to communities of gbMSM including reference to its transmission characteristics. We have also given further information around the Irish context to contextualise this study.
3. Information regarding the duration of the study and the tools used are contained in the manuscript and these were made clearer. We have given further explanation of the terms and process of critical realism and reflexive thematic analysis.
4. We have clarified that there is no census data which addresses sexual orientation in Ireland so it is difficult to comment on how representative the sample is, but that a diversity in demographics were represented. We have also given an overview of the themes as advised.
5. We have noted the types of community-engaged initiatives represented in the papers cited to support our discussion on the important role they can play. We have added in some research around how stigma has been shown to impact on HIV and sexual health testing likelihood amongst gbmsm. We have also clarified that not all responses heavily criticised the public health response from the statutory services, highlighting the need for a variety or response modes.
6. We have noted some more actionable recommendations to bring the findings forward.
7. We have retained the term ‘culturally sensitive’ as we felt it was important to specify this.
8. We have addressed some of the language and tone throughout the manuscript to help the flow.

**Reviewer 2**

1. We have addressed the stylistic inconsistencies and errors throughout the manuscript as advised
2. We have noted in the conclusion that there has been a subsequent outbreak leading to another PHEIC being announced by the WHO and contextualised this in relation to the wider discussion.
3. We have specified the sampling strategies
4. We have given a brief description of the sample (full details remain in the table)
5. We have made clear the specific quote referred to is taken from the response of a participant who had experienced mpox.

**Reviewer 3**

We have included the survey questions as an appendix.

Many thanks once again to the editorial team and to the reviewers for their attention to detail and recommendations to strengthen our paper.

We look forward to seeing the article published soon.

Yours sincerely

Yours sincerely,

_________________________

Dr John P. Gilmore RGN FHEA

Assistant Professor in Nursing
